# Minutes of the Cincinnati Local Dental Association

**Published:** 1861-01

**Authors:** T. F. Davenport

**Affiliations:** Cincinnati


### Proceedings of Societies.

### MINUTES OF THE CINCINNATI LOCAL DENTAL ASSOCIATION.

CINCINNATI, Dec. 11, 1860.

Local Dental Association met pursuant to adjournment at the Dental College. Members present—Drs. H. A. Smith, Richardson, Taft, Cameron, Wells, James and Davenport. Minutes of the previous meeting read and approved.

On motion of Dr. DAVENPORT the rules and regular order of business were suspended.

Dr. TAFT moved that hereafter the Association meet at 7 and adjourn at 9 o'clock. Adopted.

*Also*, "That a committee of two be appointed to draft a code of ethics for this Association, and to present the same at the next regular meeting for its consideration and adoption."

Resolution adopted, and Drs. Taft and H. R. Smith appointed as the committee.

Dr. TAFT also presented the following as an amendment to the Constitution:

"That the name of this Association be changed to that of the "*Academy of Dental Science.*"

Laid over for one month.

Dr. RICHARDSON mentioned a case of a lateral incisor he filled. After preparing the cavity for the filling, and drying with bibulous paper, it became flooded before any portion of the filling could be introduced. Upon examination found a considerable amount of serum in the cavity. There had been ossification of the pulp. The new formation of dentine seemed to be much harder than the other portions. Thinks it was exudation from the pulp, and must have come through a fissure between the new and old formations of dentine.

After having failed to fill with crystal gold foil, used blocks.

Dr. TAFT had seen similar cases. Thinks the exudation came through a fissure. Remembered drilling out the fissure and inserting a hickory pivot, and then filling in the ordinary manner.

Dr. RICHARDSON further thought it was a case of transformation of a portion of the pulp, and the membrane had been absorbed, leaving a fissure through which the serum exuded. Found the dentine somewhat sensitive.

Dr. TAFT mentioned a case—inferior bicuspid—pulp dead, cleaned out the tooth for filling. There was considerable *periostitis.* Used the *mallet* in condensing the filling. The sorensss very severe at first, but gradually subsided as the operation progressed, and, when the filling was completed, had been entirely removed; can fill with the mallet where the hand pressure would not succeed, owing to periostitis. Have filled many teeth where there was acute periostitis, and have had no trouble with them since; thinks the cure is permanent.

Some informal conversation was had by different members upon the fit of plates for artificial teeth. Cases were mentioned of plates fitting very well sometimes, and at others less perfectly. The difficulty was by some supposed to be owing to a modification of the circulation in the parts by the plate.

Dr. RICHARDSON explained his method of filling teeth over exposed nerves, or where there is a lamina of softened bone protecting the nerve. In the latter case, fills with osteoplasty temporarily, then removes the filling and fills with gold. In the former case (of exposed nerves), leaves a portion of the osteoplasty covering the nerve, and then fills the remainder of the cavity with gold.

Dr. TAYLOR'S paper, on the "Life and Character of Prof. C. A. Harris," not being ready, it was determined to call a meeting as soon as his paper is prepared.

Subject for discussion at next meeting—"Extraction of Deciduous Teeth."

Adjourned to meet on the second Tuesday of January, 1861. T. F. DAVENPORT, *Sec'y.*

